# Research Progress on the Anticancer Activities and Mechanisms of Polysaccharides From *Ganoderma*

**DOI:** 10.3389/fphar.2022.891171

**Published:** 2022-07-05

**Authors:** Man Wang, Fei Yu

**Affiliations:** Institute for Translational Medicine, The Affiliated Hospital of Qingdao University, College of Medicine, Qingdao University, Qingdao, China

**Keywords:** cancer, *Ganoderma*, polysaccharides, anticancer properties, chemopreventive agents, therapeutic intervention

## Abstract

Cancer ranks as a primary reason for death worldwide. Conventional anticancer therapies can cause severe side effects, and thus natural products may be promising drug candidates for cancer therapy. Accumulating evidence has verified the prominent anticancer properties of *Ganoderma* polysaccharides, suggesting that *Ganoderma* polysaccharides may be effective chemopreventive agents of natural origin. Based on their abilities to prevent cancer development by regulating the DNA damage response, cancer cell proliferation, apoptosis, host immunity, gut microbiota and therapeutic sensitivity, there has been increasing interest in elucidating the clinical implication of *Ganoderma* polysaccharides in cancer therapy. In this review, we summarize recent findings pertaining to the roles of bioactive polysaccharides from *Ganoderma* in cancer pathogenesis, discuss the multifarious mechanisms involved and propose future directions for research. A more sophisticated understanding of the anticancer benefits of *Ganoderma* polysaccharides will be helpful for improving current treatments and developing novel therapeutic interventions for human malignancies.

## 1 Introduction

*Ganoderma*, named lingzhi in China, is one of the most well-known medicinal herbs worldwide (Loyd et al., 2018). *Ganoderma* is a large, diverse and widely distributed genus in the family Ganodermataceae that comprises at least 300 species (Winska et al., 2019). The common species of *Ganoderma* include *G. applanatum*, *G. atrum*, *G. formosanum*, *G. lucidum*, *G. sinense,* and *G. tsugae* (Cao et al., 2018). *Ganoderma* is considered as the “marvelous herb” and has been widely applied for centuries as a drug or nutraceutical to treat many forms of diseases, including autoimmune diseases, diabetes, gastrointestinal disorders and hepatitis (Cao et al., 2018). Particularly, *Ganoderma* can be an alternative adjunctive therapy for cancer (Zhang Y. et al., 2019). *Ganoderma* harbors a variety of components including alkaloids, benzoic acid derivatives, glycoproteins, meroterpenoids, polysaccharides, steroids, and triterpenoids (Sohretoglu and Huang, 2018). Among these, polysaccharides are the main bioactive compound in *Ganoderma* and contribute to its health benefits (Wen et al., 2022).

The most common classes of polysaccharides from *Ganoderma* spp. are glucans, glycopeptides, glycoproteins, and water-soluble heteropolysaccharides made up of different monosaccharides linked by α-1, 3, α-1, 4, α-1, 6 or β-glycosidic bonds (Cor et al., 2018). Polysaccharides from *Ganoderma* have been widely studied for their versatile pharmacological activities, such as anticancer, anti-inflammatory, antimicrobial, antioxidant, and immunoregulatory properties (Seweryn et al., 2021). *Ganoderma* polysaccharides exert anticancer actions through various mechanisms, including impairment of the DNA damage repair pathway, inhibition of cell proliferation and migration, activation of cell death pathways, reinforcement of antitumor immunity, amelioration of gut microbiota dysbiosis and enhancement of cancer cell susceptibility to anticancer therapies. A comprehensive perception of the biological functions of *Ganoderma* polysaccharides will likely bring significant advances in cancer treatment. However, there are a very few literatures that provide an in-depth landscape of the mechanisms of action of *Ganoderma* polysaccharides in cancer. In this review, we provide an overview of the anticancer effects of bioactive polysaccharides isolated from *Ganoderma* spp., with a focus on the underlying mechanisms responsible for their activities.

## 2 The Source and Structures of Polysaccharides From *Ganoderma*

*Ganoderma* harbors 59% crude fiber, 26–28% carbohydrate, 7–8% crude protein and 3–5% crude fat (Wang J. et al., 2017). Polysaccharides from *Ganoderma* are structurally diverse biomacromolecules with molecular weights ranging from thousands to millions. So far, over 200 polysaccharides have been separated from spores, mycelia and fruiting bodies from *Ganoderma* spp., including α-D-glucans, α-D-mannans, β-D-glucans, and polysaccharide-protein complexes (Ahmad, 2018; Hwang et al., 2018). Polysaccharides have a linear to highly branched conformation (Zeidan et al., 2017). Polysaccharides from *Ganoderma* are water-soluble while insoluble in alcohol (Lu et al., 2020). Polysaccharides are mainly categorized into two types according to the count of different monomers, homopolysaccharides and heteropolysaccharides (Ferreira et al., 2015). Most polysaccharides from *Ganoderma* are heteropolysaccharide consisting of various monosaccharides including arabinose, galactose, glucose, mannose and xylose in diverse conformations, while a few are homopolysaccharide, including dextran and galactosan (Wang J. et al., 2017). The first step in the isolation of polysaccharides is pulverization of *Ganoderma*, followed by the removal of impurities such as compounds with low weights and pigments (Ren et al., 2021). Based on the distributed location and solubility of polysaccharides in cell wall of *Ganoderma*, hot water or organic solutions including dilute acid, dilute alkalis and salt solvents can be used to extract polysaccharides (Shi, 2016). Among these, hot water extraction is the most popular approach due to its convenience, ease of operation and safety. Enzymolysis, Sevag method, lead acetate precipitation, salting out, and trichloroacetic acid (TCA) precipitation are applied in the purification of polysaccharide (Zeng et al., 2019). In addition, column chromatography methods (e.g., affinity chromatography, anion exchange column chromatography and gel permeation chromatography) can also be used to remove impurities from the polysaccharides (Lu et al., 2020). The most frequent classes of polysaccharides extracted from *Ganoderma* spp. include glucans, glycopeptides, glycoproteins, and water-soluble heteropolysaccharides (Kladar et al., 2016). *Ganoderma* polysaccharides exert a broad range of pharmacological effects, including anti-inflammatory, immunostimulating and anticancer activities (Sohretoglu and Huang, 2018). It is proposed that branching conformation and solubility features may be vital parameters in determining the anticarcinogenic capabilities of *Ganoderma* polysaccharides.

## 3 Bioavailability and Metabolism of Polysaccharides

Glucans are glucose polymers that are mainly divided into α-glucans and β-glucans based on the nature of chemically glycosidic bounds as either being α- or β-linked (Cerletti et al., 2021). β-glucans are a heterogeneous group of natural polysaccharides and comprise D-glucose monomers linked by β-glycosidic bonds (Han et al., 2020). β-glucans are the main polysaccharide present in *Ganoderma* (Ren et al., 2021). The pharmacokinetic profiles of three water-soluble polysaccharides (glucan phosphate, laminarin and scleroglucan) were previously characterized following intravenous administration in rats (Rice et al., 2004). Because of the variations in branching frequency, molecular size and solution conformation, pharmacokinetics for these polysaccharides were distinct. Glucan phosphate showed a smaller volume of distribution and lower clearance, leading to an elimination half-life similar to that of laminarin and scleroglucan. In case of continual administration, plasma glucan levels at steady state were inversely correlated with clearance, and differences in clearance might cause higher plasma levels of glucans with specific physicochemical properties. The pharmacokinetics after oral administration showed differences among these three glucans (Rice et al., 2005). Pharmacokinetic investigation from rats indicated that the peak plasma concentration of glucan phosphate was reached at 4 h after oral administration. By contrast, maximum plasma contents of laminarin occurred at 3 h and again at 12 h. Plasma scleroglucan levels rapidly increased after oral administration and achieved peak concentration at 15 min and again at 3 h. The plasma level of glucan phosphate was 27 ± 3% of maximum at 24 h. There was 20 ± 7% of laminarin remained in the serum at 24 h. Unlike glucan phosphate and laminarin, scleroglucan was cleared from the systemic circulation after 12 h. The bioavailability for laminarin (4.9%) or scleroglucan (4.0%) was apparently higher than for glucan phosphate (0.5%). Vetvicka et al. (Vetvicka et al., 2007) assessed the absorption and tissue distribution of enterally administrated β-glucans in a suckling rat model. The authors revealed that the majority of β-glucans could be detected in the duodenum and stomach 5 minutes following administration. Their contents rapidly declined during first 30 min. A remarkable level of β-glucans entered the proximal intestine. The shuttling of β-glucans through proximal intestine decreased with time, accompanied by an increased amount of β-glucans in the ileum.

Further study suggests that β-glucans enter the proximal small intestine and are internalized by gastrointestinal macrophages after oral administration (Zhang et al., 2018). This may be followed by the transit of β-glucans to the bone marrow and endothelial reticular system and their breakdown within the marrow. The small biologically active β-glucan fragments are secreted by the macrophages and captured by the circulating granulocytes, monocytes, and dendritic cells. However, there is limited evidence supporting this presumptive pharmacokinetics for glucans occurred in humans. The bioavailability and mechanism of glucans in human subjects deserve further study. Despite the pharmacokinetics of some fungal polysaccharides have been determined, the bioavailability of *Ganoderma* polysaccharides remains to be investigated.

## 4 The Antitumor Activities of Polysaccharides From *Ganoderma*

A growing body of evidence has indicated the anticarcinogenic property of *Ganoderma* polysaccharides (Table 1). *Ganoderma* polysaccharides repress carcinogenesis and cancer development through their action on intracellular signaling cascades, enhancement of antitumor immunity and modulation of gut microbiota. In addition, *Ganoderma* polysaccharides augment the efficacy of anticancer therapies, stressing that *Ganoderma* polysaccharides may act as a complement to existing cancer treatments. The detailed mechanisms of action of *Ganoderma* polysaccharides are discussed in the following sections.

**TABLE 1:** The biological functions and mechanisms of *Ganoderma* polysaccharides in cancer.

Polysaccharide	Origin	Effect	Anticancer mechanism	References
GLP	*Ganoderma lucidum*	Enhance radiation-mediated growth inhibition and apoptosis promotion in hepatocellular carcinoma	Inhibit the activity of DNA repair kinases ATM and DNA-PK	Yu et al. (2017)
WSG	*Ganoderma lucidum*	Decrease the viability and mobility of lung cancer cells	Inactivate EGF- and TGF-β-relevant signaling pathways	Hsu et al. (2020)
MEs(PS-GLP)	*Ganoderma lucidum*	Exhibit cytotoxicity toward lung cancer cells	Increase GLP accumulation in tumor tissues	Guo et al. (2018)
GDNB	*Ganoderma lucidum*	Repress the proliferation and motility and facilitate the apoptosis of lung cancer cells	Alleviate the expression of proliferation- and EMT-related proteins (Ki67, PCNA, N-cadherin, vimentin, Snail); Upregulate proapoptotic proteins Bax, cleaved caspase-3 and cleaved PARP, and downregulate the anti-apoptotic protein Bcl-2	Wang et al. (2019)
GLP	*Ganoderma lucidum*	Restrain the growth and migration of prostate cancer cells	Block the oncogenic PRMT6 signaling cascade and decrease the expression of migration-associated proteins FAK and FRC	Zhao et al. (2018)
WSG	*Ganoderma lucidum*	Facilitate the apoptosis of tongue cancer cells	Promote cell apoptosis by increasing the Bax/Bcl-2 ratio; block the EGFR/Atk signaling pathway	Hsu et al. (2021)
GAP	*Ganoderma applanatum*	Suppress the proliferation, invasion and migration and enhance the apoptosis of breast cancer cells	Increase the expression of autophagy-associated markers Beclin-1 and LC3; Impede the MAPK/ERK signaling pathway	Hanyu et al. (2020)
RSGLP	*Ganoderma lucidum*	Promote the apoptosis of gastric cancer cells	Downregulate Bcl-2 and pro-caspase-3 and upregulate cleaved PARP; elevate the expression of autophagy-related proteins (LC3-II and p62)	Zhong et al. (2021)
GLP	*Ganoderma lucidum*	Reinforce p53-induced apoptosis in colorectal cancer cells	Repair p53 ability to induce the expression of the downstream pro-apoptotic proteins Bax and p21	Jiang et al. (2017)
EGLP	*Ganoderma lucidum*	Promote the apoptosis of colorectal cancer cells	Upregulate Bax, p-ERK and cleaved caspase-3; downregulate Bcl-2, p-Akt1 and COX-2	Bai et al. (2020)
GLP	*Ganoderma lucidum*	Induce early stage autophagy in colorectal cancer cells	Increase the expression of LC3-II and p62 and promote autophagosome production; prevent the autophagosome-lysosome fusion; induce the MAPK/ERK signaling cascade	Pan et al. (2019)
EGLP	*Ganoderma lucidum*	Accelerate cervical cancer cell apoptosis	Diminish the expression of Bcl-2 and COX-2 and raise the expression of Bax and cleaved caspase-3	Kong et al. (2019)
GLP	*Ganoderma lucidum*	Enhance the apoptosis and inhibit the aggressiveness of cervical cancer cells	Increase the expression of Bax, cleaved caspase-3 and cleaved caspase-9 and decrease the expression of Bcl-2; upregulate E-cadherin and downregulate N-cadherin, vimentin and Slug; retard the JAK/STAT5 signling pathway	Jin et al. (2020)
GLP	*Ganoderma lucidum*	Facilitate the apoptosis of cervical carcinoma cells	Increase Bax expression and reduce Bcl-2 expression	Zhu et al. (2019)
GLP	*Ganoderma lucidum*	Retard the viability and induce the apoptosis of prostate cancer cells	Enhance PARP cleavage and reduce the expression of pro-caspase-3, -6 and -9; suppress the MAPK/ERK signaling pathway	Wu et al. (2018)
GLP-Au	*Ganoderma lucidum*	Prevent breast cancer growth and metastasis	Exert strong immunostimulatory effects on T cell expansion via DC activation	Zhang et al. (2019a)
GLP-BiNP	*Ganoderma lucidum*	Inhibit breast cancer growth	Increase the count of intratumor CD8^+^ T cells and the ratio of IFN-γ to IL-4 in serum	Yu et al. (2019)
GLP	*Ganoderma lucidum*	Repress glioma growth	Stimulate the maturity of DCs and favor the proliferation of spleen lymphocytes	Wang et al. (2018)
GLP	*Ganoderma lucidum*	Prevent the development of lung cancer	Favor MDSC differentiation, T cell expansion and Th1 cytokine production	Wang et al. (2020)
GLP	*Ganoderma lucidum*	Inhibit melanoma growth	Reduce the count of CD68^+^ macrophages	Liu et al. (2021b)
GLP	*Ganoderma lucidum*	Suppress the proliferation of hepatocellular carcinoma cells	Promote primary macrophage polarization to M1 type; elevate the production of TGF-β1, TNF-α, IL-1β and IL-6	Song et al. (2021)
GLP	*Ganoderma lucidum*	Prevent colorectal carcinogenesis	Improve the intestinal barrier function; increase the count of SCFA-producing bacteria (e.g., *Alloprevotella rava*, *Bifidobacterium choerinum* and *Prevotella* spp.) and decrease the count of sulfate-reducing bacteria (e.g., *Desulfosporosinus* spp. and *Desulfotomaculum* spp.)	Khan et al. (2019)
GLP	*Ganoderma lucidum*	Antagonize AOM/DSS-induced colorectal carcinogenesis	Increase the ratio of Firmicutes to Bacteroidetes and enrich the population of *Bifidobacterium*, *Lactobacillus*, *Alistipes_finegoldii* and *Bacteroides_acidifaciens*; alleviate the abundance of cancer-associated genera (*Alistipes*, *Desulfovibrio*, *Lachnoclostridium*, *Oscillibacter* and *Parasutterella*), *Bifidobacterium_pseudolongum* and *Lactobacillus_reuteri*	Guo et al. (2021a)
GLP	*Ganoderma lucidum*	Inhibit the viability and mobility of breast cancer cells	Reduce the ratio of Firmicutes to Bacteroidetes, the populations of *Bacteroides* and *Lactobacillus*; increase the abundance of the SCFA-producing *Alistipe* and the tumor-suppressive *Prevotellaceae_UCG-001*	Li et al. (2018)
GSP	*Ganoderma sinense*			
SGP	*Ganoderma lucidum*	Exhibit strong anticancer activity against breast cancer	Elevate the number of *Bacteroides* and *Ruminococcus*; decrease the number of cancer-related genera *Desulfovibrio* and *Odoribacter*	Su et al. (2018)
SGP	*Ganoderma lucidum*	Alleviate the adverse effect of the chemotherapeutic agent paclitaxel	Ameliorate chemotherapy-induced intestinal barrier injury; inhibit endotoxemia and elevate the expression of tight junction proteins (E-cadherin, β-catenin, occludin and ZO-1)	Li et al. (2020a)
GL-pp	*Ganoderma lucidum*	Retard melanoma metastasis	Increase the population of *Bacteroides* and decrease the population of *Christensenellaceae*, *Desulfovibrio*, *Odoribacter* and *Parvibacter*	Xian et al. (2021)
GLP	*Ganoderma lucidum*	Improve therapeutic responsiveness of oral squamous cell carcinoma cells	Lower the expression of CSC, EMT and chemosensitivity markers (BMI1, p75NGFR, twist, N-cadherin and ABCB1)	de Camargo et al. (2022)
WSG	*Ganoderma lucidum*	Enhance lung cancer cell susceptibility to cisplatin	Augment cisplatin-induced apoptotic responses	Qiu et al. (2021)

### 4.1 Blockade of DNA Damage Repair Pathway

DNA damage response plays an important role in maintaining genomic stability (Urulangodi and Mohanty, 2020). Dysregulated DNA damage repair pathways are linked with cancer occurrence and development (Li LY. et al., 2020). Targeting DNA repair pathways could enhance cancer cell sensitiveness to anticancer therapies and is a potential therapeutic option for cancer intervention. *G. lucidum* polysaccharide (GLP) was shown to augment radiation-mediated growth inhibition and apoptosis promotion in hepatocellular carcinoma (HCC) (Yu et al., 2017). As one of mainstream anticancer regimens, radiation can induce cancer cell death by triggering DNA damage (Li LY. et al., 2020). Ataxia-telangiectasia mutated (ATM) and DNA dependent-protein kinase (DNA-PK) are responsible for the repair of radiation-induced DNA lesions and double-strand break (DSB) (Bannik et al., 2021). GLP impaired the DNA damage repair pathway in radiation-treated HCC cells by restraining the function of ATM and DNA-PK (Yu et al., 2017). The protein kinase B (Akt) signaling pathway is involved in DNA damage repair (Alemi et al., 2022). As expected, the Akt repressor increased the activities of ATM and DNA-PK and weakened the anticancer effects of GLP on HCC cells. It could be inferred that the Akt signaling pathway mediated GLP-induced HCC radiosensitization. GLP might be a promising radiation sensitizer to enhance the therapeutic effectiveness of ionizing radiation in HCC patients. Because of its antioxidant property, GLP exhibited radioprotective activity and supported DNA damage repair in human lymphocytes (Pillai et al., 2014). It is possible that there exists a complex interplay between the promotion and repair of radiation-induced DNA damage in GLP-treated cells. The DNA repairing potential of GLP may occupy a dominant position in normal cells, contributing to the prevention of genomic instability and cancer formation. Conversely, GLP facilitates cancer cell death by enhancing DNA damage. However, considerable efforts are required to clarify the knowledge gaps concerning the context-dependent effects of GLP on DNA damage repair.

**FIGURE 1:**
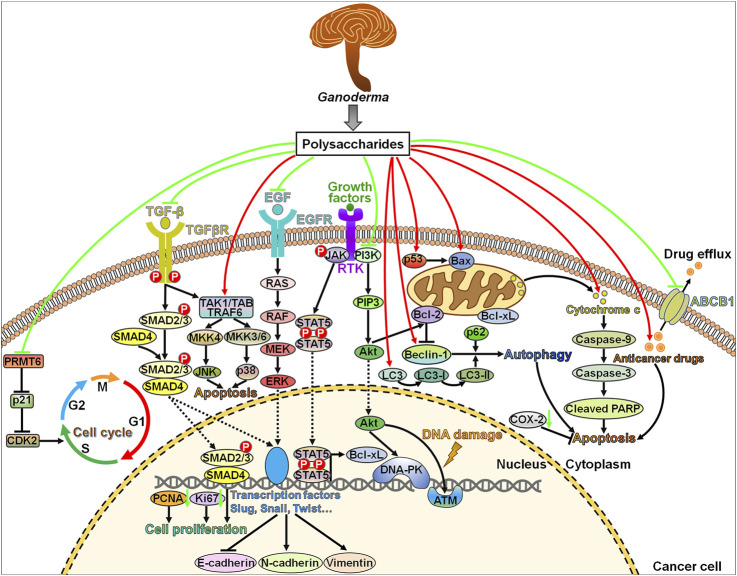
Schematic of the main anticancer molecular mechanisms of *Ganoderma* polysaccharides. *Ganoderma* polysaccharides retard cell cycle progression by targeting the PRMT6/p21/CDK2 signaling pathway. *Ganoderma* polysaccharides inhibit cancer cell proliferation by suppressing the TGF-β/SMAD2 signaling cascade or downregulating PCNA and Ki67. *Ganoderma* polysaccharides exert pro-apoptotic actions through modulation of several signal transduction cascades, including the JNK, p38 and JAK/STAT5 signaling pathways. Moreover, they can aggrandize cellular apoptotic response by altering the expression of apoptosis-relevant proteins, such as Bax, Bcl-2, caspase-9, caspase-3 and PARP. *Ganoderma* polysaccharides impede the EMT program in cancer cells by inactivating the ERK signaling pathway. Intriguingly, *Ganoderma* polysaccharides interfere with the DNA damage repair response via restricting the activities of DNA-PK and ATM through suppression of the upstream PI3K/Akt signaling cascade. *Ganoderma* polysaccharides increase the expression of Beclin-1 and LC3 to initiate early autophagic flux, which subsequently facilitates cancer cell apoptosis. In addition, *Ganoderma* polysaccharides augment the pro-apoptotic effect of chemotherapeutic drugs and lower the expression of the drug efflux pump ABCB1, which reinforce the anticancer efficacy of chemotherapeutic drugs. PRMT6, protein arginine methyltransferase 6; CDK2, cyclin-dependent kinase 2; TGF-β, transforming growth factor-β; TGFβR, transforming growth factor-β receptor; SMAD, small mother against decapentaplegic protein; PCNA, proliferating cell nuclear antigen; TAK1, transforming growth factor-β-activated kinase 1; TAB, TAK1-binding protein; TRAF6, tumor necrosis factor receptor-associated factor 6; MKK, mitogen-activated protein kinase; JNK, c-Jun N-terminal kinase; EGF, epidermal growth factor; EGFR, epidermal growth factor receptor; RAS, rat sarcoma virus; RAF, rapidly accelerated fibrosarcoma; MEK, mitogen-activated protein/extracellular signal-regulated kinase; ERK, extracellular signal-regulated kinase; JAK, Janus kinase; RTK, receptor tyrosine kinase; STAT5, signal transducer and activator of transcription 5; PI3K, phosphatidylinositol 3-kinase; PIP3, phosphatidylinositol 3,4,5-triphosphate; Akt, protein kinase B; Bcl-xL, B-cell lymphoma-extra large; DNA-PK, DNA-dependent protein kinase; ATM, ataxia-telangiectasia mutated; Bcl-2, B-cell lymphoma-2; Bax, Bcl-2-associated X protein; LC3, microtubule-associated protein light chain 3; COX-2, cyclooxygenase-2; cleaved PARP, cleaved poly (ADP-ribose) polymerase; ABCB1, adenosine triphosphate (ATP)-binding cassette subfamily B member 1.

The DNA damage repair pathway plays a pivotal role in the development of chemoresistance or radioresistance in cancer (Lagunas-Rangel and Bermudez-Cruz, 2020). Induction of DNA damage prevents cancer growth and initiates cancer cell apoptosis. Targeting the DNA damage repair pathway may enhance the efficacy of traditional therapeutic strategies. *Ganoderma* polysaccharides can target DSB repair kinases ATM and DNA-PK, thus inhibiting cancer pathogenesis (Figure 1). DSB is a major threat to genomic integrity and may arise from the presence of genotoxic agents (Karl et al., 2022). Cells adopt several DSB repair mechanisms including homologous recombination (HR), non-homologous end-joining (NHEJ) and single strand annealing (SSA) that involve a number of mediators and effectors [e.g., ATM, breast cancer susceptibility gene 1/2 (BRCA1/2), checkpoint kinase 1/2 (CHK1/2) and DNA-PK] (Blasiak, 2021). The contribution of *Ganoderma* polysaccharides to the disruption of particular DSB repair mechanisms awaits further confirmation. Cells also evolve distinct mechanisms involved in repairing other types of DNA lesions, such as replication errors and single-strand break (SSB) (Chatterjee and Walker, 2017). An in-depth investigation on the crosstalk among diverse DNA repair systems will be conducive to the development of more effective therapies that target specific repair pathways. Currently, there are very few literatures on the effect of *Ganoderma* polysaccharides on the highly intricate DNA repair machineries. Therefore, it is of great necessity to fully understand the roles of *Ganoderma* polysaccharides in the regulation of DNA repair systems.

### 4.2 Inhibition of Cancer Cell Proliferation and Migration

*Ganoderma* polysaccharides can affect cancer cell proliferation and malignancy. The water soluble polysaccharides from *G. lucidum* (WSG) significantly reduced the viability and mobility of lung cancer cells (Hsu et al., 2020). WSG impeded the phosphorylation of multiple intracellular signaling molecules [e.g., extracellular signal-regulated kinase 1/2 (ERK1/2), focal adhesion kinase (FAK), Akt and SMAD2] upon epidermal growth factor (EGF) and transforming growth factor-β1 (TGF-β1) stimulation, thereby blocking EGF- and TGF-β-activated intracellular signaling pathways (Figure 1). Mechanistically, WSG accelerated the degradation of epidermal growth factor receptor (EGFR) and transforming growth factor-β receptors (TGFβRs) by mobilizing proteasome- and lysosome-mediated protein degradation pathways. It remains to explore how WSG affects the ubiquitination of EGFR. The molecular mechanisms underlying WSG-mediated TGFβR degradation also warrant special attention. It is necessary to verify whether WSG can directly combine with TGFβRs or whether WSG promotes the transport of TGFβRs to the lysosome. Furthermore, WSG repressed lung cancer growth and metastasis *in vivo* and prolonged the survival of tumor-bearing mice (Hsu et al., 2020). WSG might act as a potential chemopreventive agent against lung cancer. Clinical studies are still required to assess the safety and effectiveness of WSG used alone or co-treatment with chemotherapeutic agents in patients with lung cancer.

Coix seed oil has been used to treat cancer in clinical practice (Wang Y. et al., 2017). GLP-integrated coix oil-based microemulsion (MEs(PS-GLP)) showed a dose-dependent cytotoxicity toward lung cancer cells (Guo et al., 2018). *In vivo* experimental studies demonstrated that MEs(PS-GLP) exhibited significant tumor-suppressing capabilities in nude mice bearing lung cancer xenografts and might be an oral anticancer agent delivery system. Particularly, the integration of GLP extended the intestinal retention of MEs (PS-GLP), which might be attributable to the enhancement of intestinal attachment caused by GLP. The connection of GLP also promoted the accumulation of MEs (PS-GLP) in tumor tissues. These results supported further studies to substantiate the therapeutic efficacy of MEs (PS-GLP) in patients with lung cancer. In addition, the mechanisms of action of MEs (PS-GLP) in tumor suppression need to be thoroughly defined.

Ganoderan B (GDNB), a component of GLP, repressed the proliferation of lung cancer cells by downregulating Ki67 and proliferating cell nuclear antigen (PCNA) (Wang et al., 2019). Moreover, GDNB inhibited lung cancer cell invasion and migration by reducing the expression of N-cadherin, vimentin and Snail and increasing the expression of E-cadherin. In addition, GDNB fostered lung cancer cell apoptosis by altering the expression levels of apoptosis-related proteins including B-cell lymphoma-2 (Bcl-2), Bcl-2-associated X protein (Bax), cleaved caspase-3, and cleaved poly (ADP-ribose) polymerase (PARP). *In vivo* experimental evidence verified that GDNB suppressed the growth and motility and promoted the apoptosis of lung cancer cells by inactivating the ERK signaling cascade in tumor-bearing mice. These findings demonstrated that GDNB had clinical implication in cancer treatment. However, only the effect of GDNB on the expression levels of marker proteins in the ERK signaling pathway was determined in this study. The involvement of the ERK signaling pathway in GDNB-mediated tumor suppression warrants additional investigation. Besides, there is still a need for further evidence with respect to the role of GDNB in lung cancer.

GLP repressed cell growth and migration, and induced cell cycle arrest in prostate cancer cells (Zhao et al., 2018). Mechanistical investigation showed that GLP impeded the oncogenic protein arginine methyltransferase 6 (PRMT6) signaling pathway and inhibited the expression of migration-associated proteins FAK and florigen repression complex (FRC). P21 is a vital downstream molecule of PRMT6 and can induce cell cycle arrest (Feng et al., 2019). Cyclin-dependent kinase 2 (CDK2) is also a key mediator of cell cycle progression and plays a crucial role in the migration and metastasis of cancer cells (Lukasik et al., 2021). PRMT6 depletion apparently increased the expression of p21 while decreased that of CDK2. Thus, GLP was effective in inhibiting the progression of prostate cancer by orchestrating cell cycle progression. Further work is required to determine whether GLP has a direct effect on the activity of these cell cycle-related proteins.

Cell cycle dysregulation and sustained cell proliferation are key hallmarks of cancer (Kappel et al., 2019). Unsurprisingly, the aberrant expression of cell cycle-relevant genes is causative to carcinogenesis (Guo H. et al., 2021). Previous studies have demonstrated that *Ganoderma* polysaccharides can prevent cancer growth by affecting the expression of cell cycle-related genes p21 and CDK2. Moreover, *Ganoderma* polysaccharides directly modulate the activities of cellular proteins and signaling cascades that are involved in cell proliferation and motility. Much work is still required to understand the detailed mechanisms by which *Ganoderma* polysaccharides regulate cancer development. Multiple intracellular signal transduction cascades may act synergistically on *Ganoderma* polysaccharide-mediated tumor inhibition. It is intriguing whether *Ganoderma* polysaccharides have an impact on the crosstalk between cancer-associated signaling pathways. The profound effect of *Ganoderma* polysaccharides on intracellular signaling networks is worthy of further study.

### 4.3 Regulation of Cell Death Pathways

Induction of cell death is currently the chief therapeutic purpose of anticancer therapies. Many studies have revealed the promotive action of *Ganoderma* polysaccharides on cell death in cancer. Reportedly, WSG strikingly decreased cell viability and colony formation of tongue cancer cells (Hsu et al., 2021). WSG promoted tongue cancer cell apoptosis by elevating the ratio of Bax to Bcl-2. WSG inhibited the phosphorylation of EGFR and Akt. WSG had a synergistic effect in combination with cisplatin on cell viability inhibition and apoptosis promotion. Particularly, WSG alleviated the cytotoxicity of cisplatin toward normal oral epithelial cells. Thus, WSG inhibited the development of tongue cancer by blocking the EGFR/Akt signaling pathway and inducing cell apoptosis. WSG might be a safe and effective adjuvant chemotherapy for tongue cancer.

The medicinal fungus *G. applanatum* polysaccharide (GAP) consisted of arabinose, fucose, glucose, and rhamnose (Hanyu et al., 2020). GAP suppressed the proliferation, invasion and migration of breast cancer cells in a time-/dose-dependent manner. GAP induced chromatin condensation and promoted apoptosis in breast cancer cells. GAP increased the expression of autophagy-associated markers Beclin-1 and microtubule-associated protein light chain 3 (LC3). The combination of GAP with chloroquine (CQ), an inhibitor of late autophagy, further increased the levels of LC3-II, suggesting that GAP stimulated early autophagy to induce breast cancer cell death. The mitogen-activated protein kinase (MAPK)/ERK signaling pathway has been associated with cell proliferation, apoptosis, autophagy and migration (Asl et al., 2021). Mechanistic investigation showed that GAP lowered the expression of phosphorylated ERK1/2 (p-ERK1/2) while increased the expression of phosphorylated p38 (p-p38) and phosphorylated c-Jun N-terminal kinase (p-JNK). Altogether, GAP exerted inhibitory roles in the development of breast cancer by favoring apoptosis and autophagy via the MAPK/ERK signaling cascade. *In vivo* experimental studies are required to confirm the anticancerous property of GAP. It is also necessary to clarify whether GAP promotes breast cancer cell apoptosis by inducing early autophagy. Further work is still warranted to ascertain whether other signaling pathways mediate the anticarcinogenic actions of GAP.

Zhong et al. Zhong et al. (2021) compared the anticancer activities of polysaccharides from sporoderm-removed spores (RSGLP) and sporoderm-broken spores of *G. lucidum* (BSGLP). RSGLP showed stronger inhibitory activity against gastric cancer cell viability than BSGLP. In terms of mechanism, RSGLP markedly enhanced the apoptosis of gastric cancer cells by downregulating Bcl-2 and pro-caspase-3 and upregulating cleaved PARP. Moreover, RSGLP increased the expression of autophagy-related proteins (LC3-II and p62). CQ further enhanced RSGLP-induced upregulation of LC3-II and p62, and the autophagy inducer rapamycin co-treatment with RSGLP elevated p62 expression. RSGLP facilitated autophagy initiation and autophagosome accumulation, and repressed autophagosome-lysosome function in gastric cancer cells. RSGLP-mediated regulation of autophagy is a highly fine-tuned process that awaits in-depth investigations. The effects of RSGLP on autophagy was partially responsible for its induction of apoptosis in gastric cancer cells. In summary, RSGLP could act as a potential autophagy suppressor in the intervention of gastric cancer.

5-Fluorouracil (5-FU) stabilized wild-type p53 in colorectal cancer (CRC) cells and thus induced apoptosis (Jiang et al., 2017). GLP alone or in combination with 5-FU suppressed CRC cell proliferation by reactivating mutant p53. GLP also restored the ability of p53 to transactivate the downstream genes encoding pro-apoptotic proteins Bax and p21. 5-FU induced the translocation of mutant p53 to the mitochondria. GLP further enhanced 5-FU-induced p53 mitochondrial translocation, caused mitochondrial membrane permeabilization (MMP) and led to cytochrome c release from the mitochondria. These events resulted in the activation of the downstream caspase cascade to initiate CRC cell apoptosis. GLP might severe as a potential chemotherapeutic agent for the treatment of cancer with mutant p53. Enzymatically hydrolyzed GLP (EGLP) showed cytotoxic activities against CRC cells (Bai et al., 2020). EGLP enhanced CRC cell apoptosis by upregulating Bax, p-ERK and cleaved caspase-3 and downregulating Bcl-2, phosphorylated Akt1 (p-Akt1) and cyclooxygenase-2 (COX-2). These findings provided scientific evidence supporting the therapeutic potential of EGLP in CRC treatment. GLP could initiate CRC cell autophagy by upregulating the levels of LC3-II and p62 and promoting the production of autophagosomes (Pan et al., 2019). Supplementation of CQ further raised the expression level of LC3-II and p62 and enhanced the accumulation of autophagosomes. GLP blocked the autophagosome-lysosome fusion through inhibition of lysosome acidification and lysosomal cathepsin activities. GLP-induced autophagosome accumulation contributed to its promotion effects on CRC cell apoptosis. The addition of 3-methyladenine (3-MA), an inhibitor of early autophagy, restrained GLP-induced cell apoptosis. On the contrary, suppression of late stage autophagy by CQ strengthened the anticancer actions of GLP. In addition, the MAPK/ERK signaling pathway was involved in GLP-induced autophagosome accumulation and apoptosis in CRC cells. Consistently, GLP apparently suppressed tumor growth and autophagy flux in a nude mouse xenograft model of CRC. These observations provide a solid basis for potential application of GLP in CRC treatment. In short, GLP possesses the ability to target diverse molecules and signaling pathways that are associated with apoptosis and autophagy in CRC. The interconnection between these signaling networks is worthy of continuous exploration.

EGLP retarded the growth of cervical carcinoma in tumor-bearing mice (Kong et al., 2019). The chemotherapeutic agent cyclophosphamide (CTX) showed cytotoxicity toward immune organs. In contrast, EGLP protected the immune organs of tumor-bearing mice. Chemotherapy-induced oxidative stress represents a critical cause of anticancer medication-associated adverse effects (Zhang et al., 2022). CTX induced oxidative stresses in tumor-bearing mice by lowering the activities of antioxidant enzymes. EGLP exerted an opposite effect on antioxidant enzymes, suggesting that EGLP could alleviate oxidative stress *in vivo*. Further study showed that EGLP downregulated the expression of Bcl-2 and COX-2 and upregulated the expression of Bax and cleaved caspase-3. These observations demonstrated that EGLP was capable of promoting cervical cancer cell apoptosis. EGLP exhibited strong anticancer potential with little side effect and held the promise as a chemotherapeutic drug for the treatment of cervical carcinoma. GLP decreased the invasive and migratory abilities of cervical cancer cells (Jin et al., 2020). GLP elevated the expression of Bax, cleaved caspase-3 and cleaved caspase-9 and reduced the expression of Bcl-2. It also enhanced the expression of E-cadherin and decreased the expression of N-cadherin, vimentin and Slug. Meanwhile, GLP lowered the expression of phosphorylated Janus kinase (p-JAK) and signal transducer and activator of transcription 5 (STAT5) in cervical cancer cells. Epithelial-to-mesenchymal transition (EMT) is a vital process in cancer progression and plays an important role in tumor stemness, metastasis and chemoresistance (Loh et al., 2019). Loss of E-cadherin and the concomitant gain of N-cadherin expression are hallmark features of the EMT process (Luu, 2021). These results suggested that GLP provoked the apoptosis of cervical cancer cells, and inhibited cell aggressiveness by retarding the EMT program. It is generally accepted that *Ganoderma* polysaccharides have the potential to be developed as an adjuvant therapy for cancer. The anticancer potency of chemotherapeutic agents in conjunction with GLP was previously investigated. It turned out that combined treatment with GLP and cisplatin was superior to cisplatin in inhibiting the growth of cervical carcinoma *in vivo* (Zhu et al., 2019). GLP/cisplatin combined therapy had little toxicological effects, and improved hepatic function and renal function in tumor-bearing mice. GLP/cisplatin promoted the apoptosis of cervical carcinoma cells by upregulating Bax and downregulating Bcl-2. This combination treatment could be used as an efficacious therapeutic option for cervical carcinoma.

GLP obviously suppressed the viability and induced the apoptosis of prostate cancer cells, as evidenced by PARP cleavage and downregulation of pro-caspase-3, -6, and -9 proteins (Wu et al., 2018). GLP increased the expression levels of non-steroidal anti-inflammatory drug-activated gene-1 (NAG-1) and its transcription factor early growth response-1 (Egr-1). GLP enhanced the promoter activity of NAG-1, suggesting that NAG-1 was transcriptionally modulated by GLP. GLP could facilitate the extracellular secretion of NAG-1 proteins. Suppression of NAG-1 expression remarkably prevented GLP-induced apoptosis and counteracted the roles of GLP in regulating PARP and pro-caspase expression. In addition, GLP suppressed Akt phosphorylation and the MAPK/ERK signaling pathway in prostate cancer cells. Therefore, the chemopreventive potential of GLP could be explained by its promotive effects on NAG-1 expression. Nevertheless, considerable research efforts are still needed to corroborate the exact function of NAG-1 in GLP-induced apoptosis and tumor suppression in prostate cancer.

The two programmed cell death pathways, apoptosis and autophagy, have become a topic of intense research in the context of cancer (Das et al., 2021). Generally, apoptosis functions as a tumor-suppressive pathway. Autophagy is an evolutionarily conserved cellular recycling mechanism that leads to internal hemostasis in response to varying environmental stimuli (Klionsky et al., 2021). Autophagy promotes the degradation of oncogenic molecules, hence inhibiting cancer onset and development. It should be noted that autophagy tends to act as a promoter of cancer cell survival under some circumstances, such as hypoxia and nutrition deprivation. Stress-activated autophagy may foster cancer cell survival by degrading cellular building blocks to supply cancer cells with nutrients. Thus, autophagy exerts opposite functions in cancer and can be classified into protective autophagy and lethal autophagy (Xie et al., 2020). Apoptosis and autophagy usually take place within the same cancer cell. Protective autophagy commonly counteracts apoptosis and contributes to a survival mechanism for cancer cells. In contrast, lethal autophagy has an antagonistic role. The effects of autophagy on cancer development may differ depending on tumor stage, tissue/cell type and extent of autophagy activity (Kaluzki et al., 2019). At present, there is limited evidence on the role of the apoptosis pathway in coordination of autophagy in cancer. The two cell death pathways are deemed to be interconnected, and elucidating the interaction between apoptosis and autophagy is essential for the improvement in the therapeutic efficacy of anticancer treatments. *Ganoderma* polysaccharides can simultaneously regulate the apoptotic and autophagic cell death pathways (Figure 1). Blockade of late autophagy enhances the pro-apoptotic activity of *Ganoderma* polysaccharides, hinting the occurrence of protective autophagy in *Ganoderma* polysaccharide-treated cancer. It has been hypothesized that the interruption of autophagy-mediated degradation may lead to the activation of apoptosis-relevant factors (e.g., caspases and p53), thereby enhancing cancer cell apoptosis (Petroni et al., 2020). Improving the anticancer effect through autophagy induction may be a potentially therapeutic option for cancer treatment. *Ganoderma* polysaccharides in combination with late autophagy inhibitors hold great promise as an effective anticancer strategy. Apoptosis and autophagy can exert synergistic, promoting or antagonistic effects in cancer (Xie et al., 2020). Nevertheless, the exact regulatory role of *Ganoderma* polysaccharides in the interaction pattern between apoptosis and autophagy deserves in-depth research.

### 4.4 Reinforcement of Antitumor Immunity

Given their prominent immunoregulatory function, it is suggestive that *Ganoderma* polysaccharides are implicated in antitumor immune responses. Polysaccharide nano-formulation can enhance stability and prolong immunoactivity of bioactive polysaccharides. Gold nanocomposites containing GLP (GLP-Au) was able to induce dendritic cell (DC) activation and enhanced the production of cytokines in DC including interleukin (IL)-1β, IL-6, IL-12, interferon-γ (IFN-γ), and tumor necrosis factor-α (TNF-α) (Zhang S. et al., 2019). GLP-Au-activated DC facilitated the proliferation of CD4^+^ and CD8^+^ T cells in murine spleen. GLP-Au could improve the function of antigen-presenting system and cellular immunity. DC activation plays a key role in cancer immunotherapy. Accordingly, GLP-Au in combination with doxorubicin markedly suppressed breast cancer growth and metastasis *in vivo*. GLP-Au co-treatment with doxorubicin increased the count of memory T cells in splenocytes from tumor-bearing mice. Moreover, GLP-Au exerted stronger immunostimulatory effects on T cell proliferation through DC activation and on memory T cell response compared with GLP. GLP-Au might have the potential to improve the efficacy of DC-based cancer immunotherapy.

GLP-conjugated bismuth sulfide nanoparticles (GLP-BiNPs) were shown to activate DCs and enhance GLP accumulation within tumor tissues of breast cancer-bearing mice (Yu et al., 2019). GLP-BiNPs in combination with radiation significantly inhibited tumor growth *in vivo* by stimulating cell apoptosis. GLP-BiNPs remodeled the tumor immunosuppression microenvironment by elevating the count of intratumor CD8^+^ T cells and the ratio of IFN-γ to IL-4 in serum. Particularly, GLP conjugation could reduce kidney injury caused by bare BiNP, which might be attributable to the anti-inflammatory benefit of GLP. GLP connection induced the radiosensitivity and antitumor immune responses in breast cancer cells, and reduced BiNP nephrotoxicity. The incorporation of immunoactive polysaccharides into nanoradiosensitizer might have the potential to aggrandize the safety and efficacy of nanoparticle-based anticancer therapeutics.

GLP elevated the levels of serum IL-2, TNF-α, and IFN-γ and augmented the cytotoxic activity of natural killer (NK) cells and T cells in glioma-bearing rats (Wang et al., 2018). IL-2, TNF-α, and IFN-γ act as critical participants in the development of antitumor immune responses. These cytokines might mediate the immune activating effect of GLP. GLP was capable of inducing the maturity of DCs and favored the proliferation of spleen lymphocytes. As a result, GLP suppressed glioma growth and prolonged the survival of tumor-bearing rats. Notably, high concentrations of GLP might result in excessive infiltration of both immunoactive and immunosuppressive cells, hence contributing to inflammation and cancer development. Additional efforts are necessary to determine the optimal dose or dose-range of GLP for cancer treatment. GLP might emerge as a potential treatment option for the immunotherapy of glioma.

GLP prevented the development of lung cancer in tumor-bearing mice (Wang et al., 2020). Mechanistically, GLP promoted the differentiation of myeloid-derived suppressor cells (MDSCs) and inhibited MDSC accumulation in spleen and tumor tissues through the caspase recruitment domain-containing protein 9 (CARD9)/nuclear factor-κB (NF-κB)/indoleamine 2,3-dioxygenase (IDO) signaling pathway. Consequently, GLP enriched the population of CD4^+^ and CD8^+^ T cells and enhanced the production of type 1 T helper (Th1)-type cytokines (IFN-γ and IL-12) in the spleen of tumor-bearing mice. The anticancer action of GLP was attributable to its induction of antitumor immune responses. The accumulation of MDSCs is a main inhibitor of the immune system, which hinders effective immunotherapeutic measures. Blockade of MSDC deposition may be an attractive approach for cancer intervention. GLP can stimulate antitumor immune responses by clearance of MDSCs, which demonstrates that GLP may be a MDSC-targeting drug in chemotherapy.

CD68^+^ macrophages have the ability to secrete inflammatory growth factors to facilitate cancer progression (Chen et al., 2017). GLP decreased the count of CD68^+^ macrophages in melanoma tissues and inhibited melanoma growth *in vivo*, thus prolonging the survival rate of tumor-bearing mice (Liu H. et al., 2021). GLP might represent an effective therapeutic option for the treatment of melanoma. GLP may cause autophagy in CD68^+^ macrophages or transform CD68^+^ macrophages to other phenotypes. However, the exact mechanism responsible for GLP-associated reduction in CD68^+^ macrophages needs to be illuminated in future studies. It was reported that GLP fostered primary macrophage polarization to M1 type and increased the production of TGF-β1, TNF-α, IL-1β, and IL-6 (Song et al., 2021). GLP-intervened macrophages evidently repressed HCC cell proliferation by inducing cell cycle arrest and activating the mitochondrial apoptosis pathway via the PI3K/Akt signaling cascade. Therefore, GLP induced antitumor immunity and favored HCC cell apoptosis by stimulating macrophages. GLP might be a promising anticancer agent for HCC treatment. The roles of GLP in altering macrophage polarity and restraining HCC progression remain to be validated *in vivo*.

**FIGURE 2:**
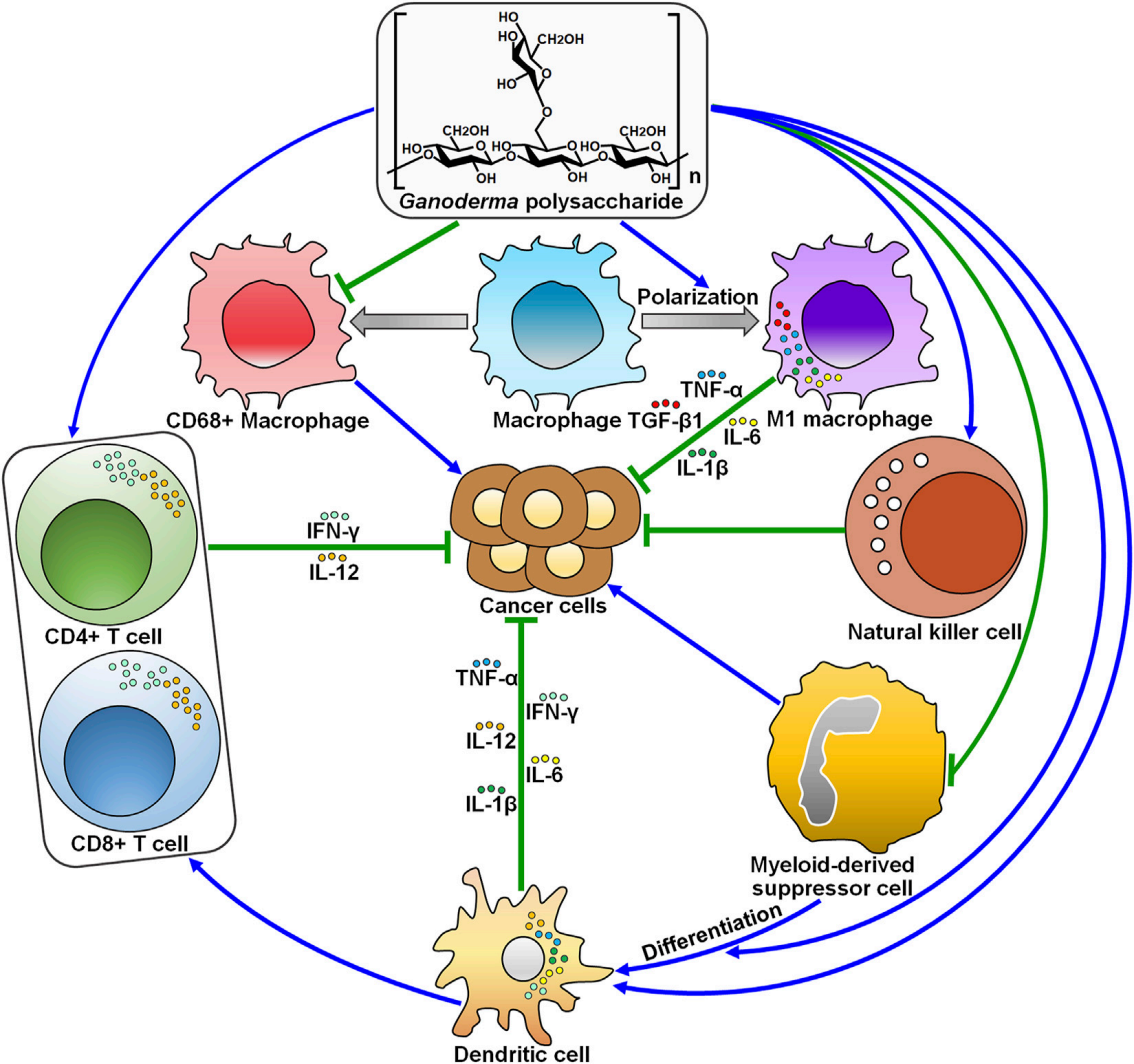
The roles of *Ganoderma* polysaccharides in modifying antitumor immunity. *Ganoderma* polysaccharides inhibit the proliferation of pro-inflammatory CD68^+^ macrophages, while they promote macrophage polarization toward the antitumoral M1 phenotype. *Ganoderma* polysaccharides can enhance the cytotoxic activity of natural killer cells. *Ganoderma* polysaccharides suppress the accumulation of myeloid-derived suppressor cells and foster their differentiation into mature dendritic cells. On the other hand, *Ganoderma* polysaccharides directly activate dendritic cells and favor the production of cytokines (e.g., IL-1β, IL-6, IL-12, IFN-γ, and TNF-α). Dendritic cells then present cancer-specific antigens to CD4^+^ T cells, thus recruiting CD8^+^ T cells to eliminate cancer cells. IFN-γ, interferon-γ; IL-12, interleukin-12; TNF-α, tumor necrosis factor-α; TGF-β1, transforming growth factor-β1; IL-6, interleukin-6; IL-1β, interleukin-1β.

GLP has been used as an adjuvant agent to strengthen the immunity of human body in clinical practice. The infiltration of activated immune cells in tumors partially contributes to better prognosis in cancer patients. Beyond cancer cells, the tumor microenvironment is a complex ecosystem that encompasses a diversity of non-epithelial cells that make up the blood vasculature (e.g., endothelial cells and smooth muscle cells), immune cells (e.g., lymphocytes, macrophages and mast cells) and stromal cells (Labani-Motlagh et al., 2020). Undoubtedly, the dynamic interaction between cancer cells and other cells within the tumor microenvironment controls cancer development and progression. Cancer cells create an immunosuppressive niche with diverse mechanisms to escape immune surveillance, such as blockade of antigen presentation, suppression of T cell function and activation of immune tolerance-related pathways (Asiry et al., 2021). Immune cells and their released mediators have emerged as a double-edged sword in cancer. It is unambiguously accepted that immune scrutiny is necessary for tumor growth inhibition. On the contrary, excessive immune responses exert a promotive role in cancer growth and metastasis. Within the tumor microenvironment, CD4^+^ and CD8^+^ T cell-mediated Th1 immune responses act as the dominant antitumor immune mechanisms (Dai et al., 2021). Oppositely, MDSCs, type 2 tumor-associated macrophages (TAMs) and their secreted cytokines act as tumor promoters. Accumulating evidence has indicated that GLP activates antitumor Th1 immune responses and impedes macrophage-mediated tumor immune evasion (Figure 2). These findings underlie the theoretical feasibility for subsequent studies of GLP as an immune adjuvant. The exploitation of effective drugs that target the immunoregulatory factors present in the tumor microenvironment would be a promising research direction. Therefore, significant research efforts should be dedicated to revealing the influence of GLP on the tumor microenvironment. In addition, it remains to ascertain whether GLP affects the reciprocal and continuous interactions between cancer cells and the tumor microenvironment.

### 4.5 Interaction With Gut Microbiota

*Ganoderma* polysaccharides remain indigestible until they reach the intestine. Polysaccharides could be metabolized by intestinal microbes and change the composition of gut microbiota. GLP improved the intestinal barrier function of Apc^*Min*/+^ mice by decreasing polyps, inducing the transition of colonic M1 to M2 macrophages, elevating E-cadherin/N-cadherin ratio and reducing oncogenic signaling molecules (e.g., p-ERK, p-AKT, p-STAT3, Src, and iNOS) (Khan et al., 2019). GLP could increase the abundance of short-chain fatty acid (SCFA)-producing bacteria (e.g., *Alloprevotella rava*, *Bifidobacterium choerinum* and *Prevotella* spp.). SCFAs have been found to play an anticarcinogenic role in CRC (Sivamaruthi et al., 2020). SCFAs regulate host cellular responses through the activation of SCFA-sensing G-protein coupled-receptors (GPCRs) and the suppression of histone deacetylases (HDACs) (Parada Venegas et al., 2019). As expected, GLP attenuated the expression of HDACs, and increased the expression of GPCRs and their relevant signaling molecules including the anticancer peptide tyrosine-tyrosine (PYY) and peroxisome proliferator-activated receptor γ (PPARγ) (Khan et al., 2019). GPCRs are able to transduce signals through attaching to PPAPγ (Wang et al., 2015). PPAPγ then alters intestinal immunity by converting the pro-inflammatory M1 to an anti-inflammatory M2 phenotype. Moreover, GLP decreased the abundance of sulfate-reducing bacteria (e.g., *Desulfosporosinus* spp. and *Desulfotomaculum* spp.) that had been associated with cancer pathogenesis. Altogether, GLP antagonized colorectal carcinogenesis by regulating the gut microbiota and host immune responses. GLP possessed the potential to be exploited as an adjuvant treatment for CRC.

GLP was capable of repressing azoxymethane (AOM)/dextran sulfate sodium (DSS)-induced colitis and colorectal carcinogenesis in mice (Guo C. et al., 2021). Mechanistically, GLP reversed gut microbiota dysbiosis caused by AOM/DSS. Firmicutes may exert an anti-CRC role, while Bacteroidetes has a close relationship with cancer development. *Bifidobacterium* and *Lactobacillus* encompass probiotic strains for CRC prevention and therapy. GLP increased the ratio of Firmicutes to Bacteroidetes and enriched the population of *Bifidobacterium* and *Lactobacillus*. Conversely, GLP diminished the abundance of cancer-associated genera, including *Alistipes*, *Desulfovibrio*, *Lachnoclostridium*, *Oscillibacter* and *Parasutterella*. GLP also decreased the amount of *Bifidobacterium_pseudolongum* and *Lactobacillus_reuteri* while raised that of *Alistipes_finegoldii* and *Bacteroides_acidifaciens*. The metabolites of intestinal microbes including SCFAs play a critical role in preserving intestinal homeostasis. The fecal levels of SCFAs including acetate, butyrate and propionate were reduced in AOM/DSS-treated mice, whereas GLP reversed their downregulation (Guo C. et al., 2021). Besides, GLP treatment markedly elevated the expression of G protein-coupled receptor 43 (GPR43) in colonic cells that specifically recognized SCFAs. It was thus proposed that GLP induced anti-inflammatory and anticarcinogenic effects by increasing GPR43 expression in response to altered intestinal microbiota composition and elevated SCFA production. Therefore, GLP inhibited AOM/DSS-elicited colorectal carcinogenesis by altering gut microbiota. The detailed mechanisms of GLP in coordination of gut microbiota are worthy of in-depth study. It is also essential to better understand how varied gut microbiota adapted by GLP affects CRC progression.

Firmicutes and Bacteroidetes are two ubiquitous phyla in mammalian gut. The increased ratio of Firmicutes to Bacteroidetes was reported to correlate with breast carcinogenesis (Wu et al., 2020). GLP and polysaccharides from *G. sinense* (GSP) reversed this tendency, contributing to tumor suppression (Li et al., 2018). They also reduced the populations of *Bacteroides* and *Lactobacillus*, while increased the abundance of the SCFA-producing *Alistipe* and the tumor-suppressive *Prevotellaceae_UCG-001*. Consistently, *in vivo* studies verified that GLP and GSP exerted inhibitory effects on tumor growth in breast cancer-bearing mice. Despite these intriguing results, the genuine contribution of altered gut microbiota to *Ganoderma* polysaccharide-induced tumor suppression requires further evidence for verification.

Polysaccharide derived from spore of *G. lucidum* (SGP) relieved paclitaxel (PTX)-induced gut microbiota dysbiosis by elevating the count of *Bacteroides* and *Ruminococcus* and reducing the count of cancer-related genera *Desulfovibrio* and *Odoribacter* (Su et al., 2018). Meanwhile, the combination of SGP and PTX showed synergistic effects against breast cancer *in vivo*. Collectively, SGP restored gut microbiota dysbiosis and thus alleviated adverse effects of chemotherapy, suggesting its potential implication as a supplement for the treatment of breast cancer. Another study revealed that SGP relieved PTX-caused body weight lost and ameliorated PTX-induced intestinal barrier injury in breast cancer-bearing mice (Li D. et al., 2020). The combined administration of SGP and PTX could improve intestinal barrier function by suppressing endotoxemia and upregulating tight junction proteins, such as E-cadherin, β-catenin, occludin and ZO-1. SGP conferred protection against PTX-induced apoptosis in intestinal cells by restraining microtubule polymerization. Collectively, SGP could repair PTX-induced small intestinal barrier injury, stressing its clinical implication as an adjunctive chemotherapy for breast cancer. The potential of *Ganoderma* polysaccharides as protective agents in cancer treatment has been increasingly acknowledged. Further exploration of the interaction between *Ganoderma* polysaccharides and gut microbiota will be helpful in disclosing the mechanisms associated with protective benefits offered by *Ganoderma* polysaccharides during chemotherapy.

*G. lucidum* polysaccharide peptide (GL-pp) markedly inhibited sleep fragmentation-induced tumor metastasis in melanoma-bearing mice (Xian et al., 2021). GL-pp altered the composition of gut microbiota in melanoma-bearing mice by increasing the number of *Bacteroides* and decreasing the number of *Christensenellaceae*, *Desulfovibrio*, *Odoribacter,* and *Parvibacter*. GL-pp promoted macrophage polarization and increased the serum level of TNF-α. GL-pp exhibited antimetastatic activities against breast cancer partially through mitigation of gut microbiota dysbiosis. It is obscure whether GL-pp has similar effects on intestinal dysbiosis in cancer patients, which could be a subject of future studies.

**FIGURE 3:**
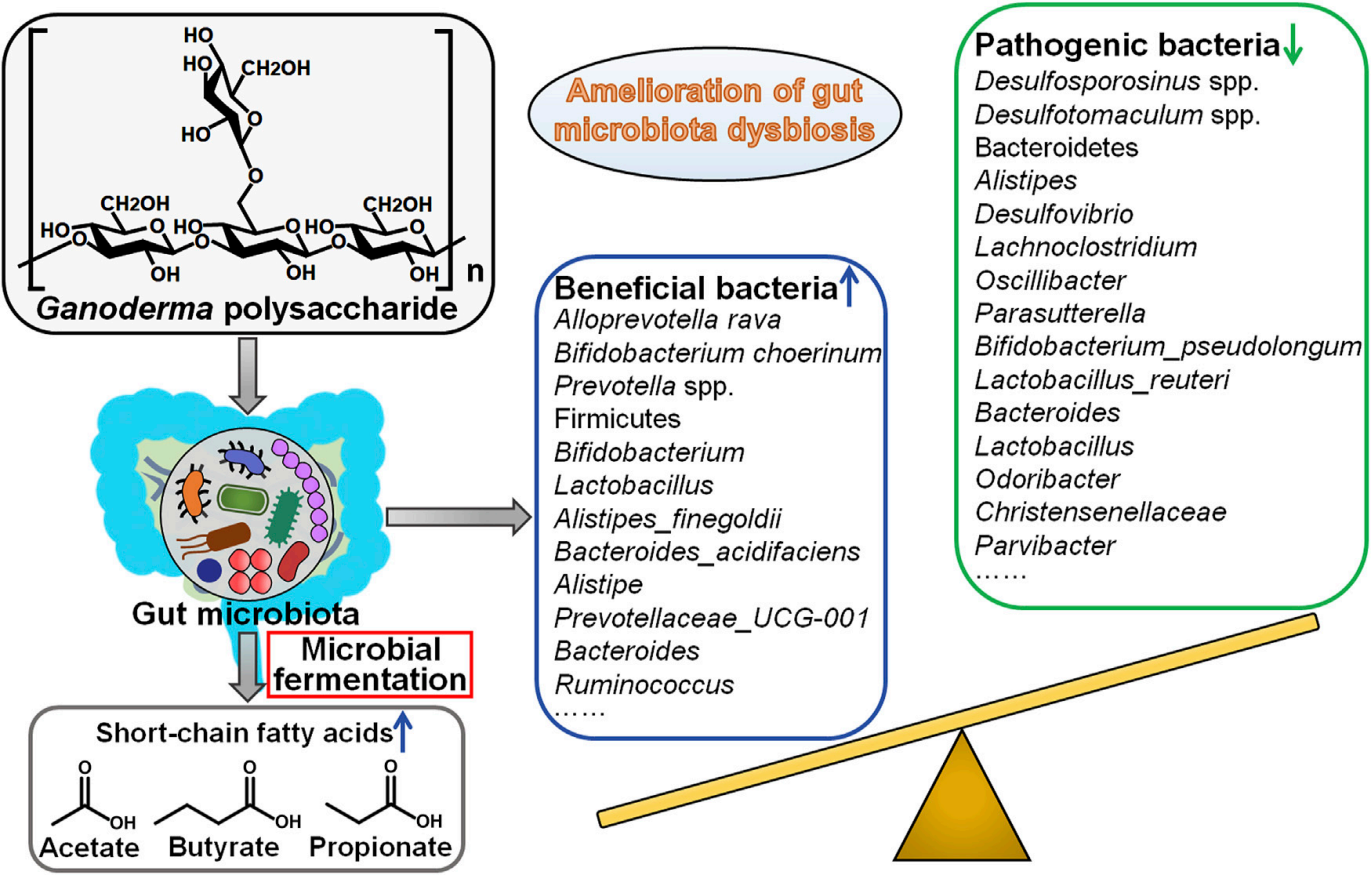
Mechanistic implications of intestinal microbiota in *Ganoderma* polysaccharides’ protective effects against cancer. Gut microbiota dysbiosis has been associated with carcinogenesis and cancer development. *Ganoderma* polysaccharides can affect gut microbiota composition. Particularly, *Ganoderma* polysaccharides enrich beneficial commensal microorganisms (e.g., *Alloprevotella rava*, *Bifidobacterium choerinum* and *Prevotella* spp.) while diminish the quantity of pathogenic microbial species (e.g., *Desulfosporosinus* spp., *Desulfotomaculum* spp. and Bacteroidetes), thereby leading to the amelioration of gut microbiota dysbiosis and tumor inhibition. Furthermore, *Ganoderma* polysaccharides increase the abundance of short-chain fatty acid (SCFA) producers and enhance the generation of SCFAs, which have been found to possess anticancerous activities.

Gut microbiota is a diverse community of microbes that preserve intestinal homeostasis (Fan et al., 2021). Gut microbiota plays dual roles in cancer formation. On the one hand, certain intestinal microbes may expand during pathological dysbiosis and induce inflammation and carcinogenesis via various mechanisms including production of pro-carcinogenic toxins, regulation of cellular survival, proliferative and invasive pathways, elicitation of oxidative stress, promotion of tumor immune escape (Torres-Maravilla et al., 2021). On the other hand, beneficial bacteria, also named probiotics, can produce a variety of metabolites capable to prevent tumor genesis (Vivarelli et al., 2019). Probiotics also stimulate anticancer immune responses, foster cancer cell apoptosis and prevent chronic inflammation. The bidirectional interplay between gut microbiota and host immune system has been correlated with cancer development. At present, gut microbiota has become a new frontier in cancer research. Nevertheless, the causal relationship between gut microbiota and cancer pathogenesis needs to be further defined. Increasing knowledge about the linkage between gut microbiota and cancer will open novel perspectives for alternative therapeutic strategies for cancer prevention and treatment. Accumulating evidence has demonstrated that *Ganoderma* polysaccharides are able to reverse the dysbiosis of gut microbiota, facilitate the production of anticancerous metabolites from commensal gut microbes, inhibit pro-inflammatory responses and enhance immune surveillance (Figure 3). The studies concerning the roles of *Ganoderma* polysaccharides in regulation of gut microbiota are just the tip of the iceberg. Continual investigations are needed to comprehensively characterize *Ganoderma* polysaccharide-induced changes in gut microbiota composition and associated metabolic profile. Gut microbiota can affect the pharmacokinetics, efficacy and toxicity of chemotherapeutic agents (Wilson and Nicholson, 2017). Manipulation of gut microbiota may afford opportunities to improve clinical outcomes of cancer patients. The impact of *Ganoderma* polysaccharides on drug metabolism through regulation of gut microbiota would be an important research area that is worthy of more attention. Gut microbiota is considered to have a profound impact on host immune system (Gopalakrishnan et al., 2018). However, it has yet to be determined what composition of gut microbiota boosts anticancer immune responses. It is imperative to clarify the contribution of different members of gut microbiota to altering host immune reactions. An increasingly sophisticated comprehension of the immunoregulatory function of gut microbiota is a vital prerequisite for the development of gut microbiota-targeting anticancer therapeutic approaches. Collectively, *Ganoderma* polysaccharides remodel gut microbiota to strengthen antitumor immunity and thus may be exploited as pharmaceuticals capable to enhance therapeutic actions of cancer immunotherapy.

### 4.6 Enhancement of Therapeutic Sensitivity

Cancer cells usually develop resistance to chemotherapeutic agents, which make it arduous to eradicate tumors. The presence of cancer stem cells (CSCs) is responsible for tumor chemoresistance and recurrence (Gaggianesi et al., 2021). GLP suppressed the proliferation, metabolic activity and migration of oral squamous cell carcinoma (OSCC) (de Camargo et al., 2022). Importantly, GLP impeded CSC properties and eventually enhanced the therapeutic responsiveness of OSCC cells by lowering the expression of CSC, EMT and chemosensitivity markers (e.g., BMI1, p75NGFR, twist, N-cadherin, and ABCB1) (de Camargo et al., 2022). GLP might act as therapeutic co-adjuvants to promote CSC elimination, hence suppressing OSCC malignancy and chemosensitizing OSCC cells to traditional treatments.

It is known that cisplatin-based chemotherapy is one of the most prevalent treatments for lung cancer. A recent study revealed that the combined administration of WSG and cisplatin exerted synergistic inhibitory effects on the growth and metastasis of lung cancer *in vitro* and *in vivo* (Qiu et al., 2021). WSG potentiated cisplatin-induced apoptotic responses in lung cancer cells but alleviated its cytotoxicity toward macrophages and normal lung fibroblasts. This result implied that WSG had distinct roles in cisplatin-exposed lung cancer and normal cells. WSG may respond to different signals in cancerous and normal cells, or only respond to a specific signal in cancer cells. The mechanisms determining the fate of WSG-treated cells need to be further elucidated. Taken together, WSG augmented the anticarcinogenic activity of cisplatin against lung cancer, suggesting that WSG could be a potential adjuvant or dietary supplement to increase the effectiveness of cisplatin-based chemotherapy in patients with lung cancer.

*Ganoderma* polysaccharides and current treatments have synergistic anticancer roles. *Ganoderma* polysaccharides exert a promoting effect on the tumor-suppressive activity of chemotherapeutic agents and may act as potential chemosensitizing agents for cancer. The acquirement of cancer chemoresistance has become a major hurdle in effective cancer treatment. The development of chemoresistance involves complex mechanisms, such as promotion of DNA repair, regulation of drug efflux and metabolism, suppression of cell apoptosis, activation of cellular survival signaling cascades and enhancement of cancer stemness (Liu C. et al., 2021). Current evidence supports the bioactivity of *Ganoderma* polysaccharides in inhibiting drug efflux pump in cancer. It is still unclear whether *Ganoderma* polysaccharides interfere with other chemoresistance-associated mechanisms. Much more work is necessary to delve into the mechanisms of action of *Ganoderma* polysaccharide in regulation of cancer cell chemosensitivity.

## 5 Summary and Perspectives

*Ganoderma* is a highly appreciated vitality-promoting medical herb for over 2000 years. *Ganoderma* polysaccharides are one of the most pharmacologically active components. Our knowledge regarding the biological actives of *Ganoderma* polysaccharides is continually expanding. *Ganoderma* polysaccharides have a wide range of pharmacological activities, including anticarcinogenic, antidiabetic, anti-inflammatory, antimicrobial, and immunoregulatory actions (Seweryn et al., 2021). *Ganoderma* polysaccharides are capable of suppressing cancer development and progression through blockade of the DNA damage repair pathway, repression of cancer cell proliferation and malignancy, alternation of cell death pathways, reinforcement of antitumor immunity, restoration of gut microbiota homeostasis and potentiation of chemotherapy-mediated cytotoxicity toward cancer cells. In a randomized, double-blind placebo-controlled crossover study, 42 healthy subjects were divided into two groups that received placebo or GLP, respectively (Chiu et al., 2017). It turned out that GLP exhibited antioxidative, anti-aging and hepatoprotective efficacy in healthy volunteers by controlling oxidative stresses. Thus, *Ganoderma* polysaccharides have significant clinical value due to their anticancer efficacy and low toxicity. *Ganoderma* polysaccharides can diminish adverse reactions caused by chemotherapy. GSP has been approved as an adjuvant therapeutic agent in China for ameliorating chemotherapy- or radiotherapy-elicited leukopenia and hematopoietic damage (Zhang Y. et al., 2019). Future researches defining the clinical implication of *Ganoderma* polysaccharides as anticancer adjuvant therapies may contribute to an improvement in therapeutic options for cancer treatment.

Nevertheless, several issues must be addressed before translating promising *Ganoderma* polysaccharide-based therapies to cancer patients. First, given that *Ganoderma* polysaccharide is a mixture of glycans, the molecular mechanisms of action of different glycan components in cancer deserve further investigation in future studies. An in-depth investigation into *Ganoderma* polysaccharides could facilitate the development of efficacious chemotherapeutic agents of natural origin. Second, supplementation with *Ganoderma* polysaccharides poses a potential risk of interplay with conventional chemotherapeutic drugs. The combination of chemotherapy and immunotherapy has become a research hotspot in clinical cancer treatment. Available therapies show limited effectiveness, low tolerance and severe adverse effects in cancer patients. Thus, more effective agents are needed for chemotherapeutic strategies in cancer patients. *Ganoderma* has emerged as a valuable source of nutraceuticals with great therapeutic potential. Interest has been increasing in the use of *Ganoderma* related natural products combined with conventional therapies in cancer management. A growing body of evidence has revealed the efficacy of *Ganoderma* polysaccharides combined with anticancer agents in cancer treatment. Anticancer agents perform their functions by inducing DNA damage and cell apoptosis. Some chemotherapeutic agents contribute to an excessive accumulation of reactive oxygen species (ROS) via induction of ROS generation or inhibition of their clearance in cancer cells (Idelchik et al., 2017). Chemotherapeutic agent-induced apoptosis can be repressed by components that retard ROS production. *Ganoderma* polysaccharides are effective in scavenging ROS and protect cellular DNA from oxidative damage (Hu et al., 2019). The antioxidative activity of *Ganoderma* polysaccharides has been correlated with its potential implication as an anticancer agent. It is possible that *Ganoderma* polysaccharides could antagonize DNA damage-inducing activity of chemotherapeutic agents in cancer cells by acting as ROS scavengers. The precise function of *Ganoderma* polysaccharides in DNA damage response needs to be fully deciphered. The intricate interaction between *Ganoderma* polysaccharides and anticancer agents remains largely equivocal, and thus more researches on this topic require to be undertaken. Third, clinical evidence for the anticancer efficacy of *Ganoderma* polysaccharides is limited so far. The anticancerous actions of *Ganoderma* polysaccharides have been substantiated in experimental studies. Even with encouraging published results, follow-up efforts should be put into evaluating the therapeutic benefits of *Ganoderma* polysaccharides in cancer patients. Particularly, their optimal dosage, duration of action, safety, pharmacokinetic profile and interaction with administrated anticancer agents need to be comprehensively explored in properly designed randomized clinical trials. Moreover, it is essential to confirm whether *Ganoderma* polysaccharides are effective in treating all kinds of cancers or they only exhibit anticancer effects on specific cancers. In addition, rational clinical combination strategies, the sequence of drug administration and the appropriate time of supplementation of *Ganoderma* polysaccharides to therapy regimens should be taken into account. Although there are few clinical trials employing *Ganoderma* polysaccharide-based natural therapies, it can be concluded that they possess the advantage in boosting the therapeutic efficacy of conventional treatments and improve the quality of life in cancer patients.

*In vitro* and *in vivo* experimental studies have revealed that *Ganoderma* polysaccharides present anti-proliferative, anti-migratory, pro-apoptotic and immunomodulatory activities in cancer. These bioactive compounds also prevent cancer pathogenesis by reversing intestinal dysbiosis to trigger a variety of reactions, such as inhibition of chronic inflammation, production of antineoplastic metabolites, restoration of intestinal barrier function and potentiation of antitumor immunity. Importantly, *Ganoderma* polysaccharides can aggrandize the anticancer potential of mainstream therapies and alleviate their adverse effects. Therefore, *Ganoderma* polysaccharides may be effective and safe chemotherapeutic agents against cancer. However, future studies are warranted to completely elucidate the anticarcinogenic mechanisms of *Ganoderma* polysaccharides as well as their clinical potency as an adjunctive cancer treatment.
